# Endoscopic Image-Based Prediction of Esophageal Stenosis after ESD Using Mucosal Defect Metrics

**DOI:** 10.1245/s10434-025-18037-7

**Published:** 2025-08-16

**Authors:** Wei Lin, Yi-zhi Liang, Dun-huang Peng, Jun-xi Wang, Si-fu Huang, Jing-jing Wei, Jiang-mu Chen, Wen-ming Liu, Tai-yong Fang, Ze-hao Zhuang

**Affiliations:** 1 Department of Endoscopy Center, The First Affiliated Hospital, Fujian Medical University, Fuzhou, China; 2 Department of Endoscopy Center, National Regional Medical Center, Binhai Campus of the First Affiliated Hospital, Fujian Medical University, Fuzhou, China; 3 Department of Gastroenterology, The Second Affiliated Hospital, Fujian Medical University, Quanzhou, China

**Keywords:** Esophageal stenosis, Endoscopic submucosal dissection, Accurate measurement, Lesion area, Lesion perimeter

## Abstract

**Background:**

Stricture is a major adverse event following esophageal endoscopic submucosal dissection (ESD). Although postoperative esophageal stenosis after ESD is clearly associated with large mucosal defects, there are no quantitative criteria for the degree of the defect. We aimed to examine the predictive factors associated with the development of esophageal stenosis after ESD, and to explore quantitative indicators for predicting postoperative stenosis.

**Patients and Methods:**

A retrospective analysis of endoscopic data from patients with esophageal ESD was conducted. The area and perimeter of the resected mucosa defect were measured by Image Pro Plus 6.0. Logistic regression, receiver operating characteristic (ROC) curve, and nomogram model were adopted for analysis.

**Results:**

The median area of resected mucosal defect was 527.74 mm^2^ (IQR 322.80–823.48), the median perimeter was 91.75 mm (IQR 71.69–118.32), and the median circumferential ratio was 33% (IQR 20–40). Owing to collinearity between the perimeter and area of the resected mucosal defect, these variables were analyzed individually in the multivariate analysis (*r* = 0.958, *P* < 0.001). All three factors mentioned above were identified as independent risk factors for postoperative esophageal stenosis (*P* < 0.05), while intraoperative muscularis propria injury was not (*P* > 0.05). On the basis of the above results, prediction models were constructed and validated internally. The concordance statistics (C-statistics) of the training and validation sets including perimeter or area were 0.875 and 0.830 and 0.747 and 0.835, respectively. The Hosmer–Lemeshow goodness-of-fit test results were not significant (*P* > 0.05).

**Conclusions:**

Accurate quantification of the area and perimeter of resected mucosa by image analysis technology can accurately predict esophageal stenosis after ESD.

**Supplementary Information:**

The online version contains supplementary material available at 10.1245/s10434-025-18037-7.

Endoscopic submucosal dissection (ESD) is a common treatment for esophageal mucosal lesions.^1^ However, extensive mucosal resection often results in postoperative esophageal stenosis. This condition affects about 90% of patients who undergo resection of up to three-quarters of the esophageal circumference. In cases of full circumferential resection, stenosis is almost inevitable.^2^ Several methods, such as corticosteroid administration, endoscopic balloon dilation, and tissue sheet covering, have been used to prevent and treat esophageal stenosis after ESD. Nevertheless, some patients still experience persistent esophageal stenosis and subpar therapeutic outcomes post-ESD. Previous studies have demonstrated a correlation between postoperative stenosis and muscularis propria injury (MPI), as well as the horizontal and longitudinal dimensions of mucosal defects and the width of the remaining mucosa after esophageal ESD.^3–7^ Previous studies have only used semiquantitative methods to assess the extent of esophageal mucosal defects. No publications have quantitatively measured the exact area and perimeter of these defects. In this study, we utilized image analysis technology to quantify the area and perimeter of esophageal mucosal defects for the first time. We also conducted a semiquantitative analysis of the circumferential ratio of these defects. The purpose of this study was to identify risk factors contributing to the formation of esophageal stenosis after esophageal ESD.

## Patients and Methods

### General Information

Data from patients who underwent esophageal ESD at the First and Second Affiliated Hospitals of Fujian Medical University was collected retrospectively from January 2018 to June 2023. The data, collected via the endoscopic image acquisition system, includes post-ESD operation endoscopic reports and specimen images taken after the operation. The inclusion criteria for this study were: (1) the treatment of esophageal mucosal lesions with ESD and (2) complete case reports and clear specimen photographs. The exclusion criteria were: (1) patients who underwent additional surgical procedures or chemoradiotherapy; (2) patients who underwent ESD for submucosal tumors; (3) incomplete case reports: postoperative specimens or wound pictures were missing or the injury of proper muscularis was not recorded; (4) unclear specimen images: the specimen’s edge appeared indistinct, or the color contrast between the specimen and the background plate was insufficient for precise edge delineation; and (5) cases where a coated stent was used to prevent stenosis. Esophageal stenosis requiring intervention was defined as the inability for endoscopes with a diameter greater than 9.8 mm to pass through. The primary intervention methods included balloon dilatation, electrocoagulation incision, and local injection of triamcinolone. Some patients whose resection encompassed more than two-thirds of the esophageal circumference received prophylactic antistenosis treatment. Specifically, 5 mg/ml of triamcinolone acetonide was injected locally (immediately after ESD, multipoint injection into the wound and marginal mucosa, dosage of 1.0 ml per point, and total dose of triamcinolone acetonide of approximately 40 mg), and/or prednisolone tablets were orally administered (starting from day 3 after operation with an initial dosage of 30 mg/day for 2 weeks, then reduced to 25 mg/day for 2 weeks, and finally reduced by 5 mg per week lasting for a total of 8 weeks).^8^ All ESD procedures were only conducted by endoscopists holding the title of associate professor or above.

### Methods

#### Measurement of Mucosal Defect Area, Perimeter, and Circumferential Ratio

**Fig. 1:**
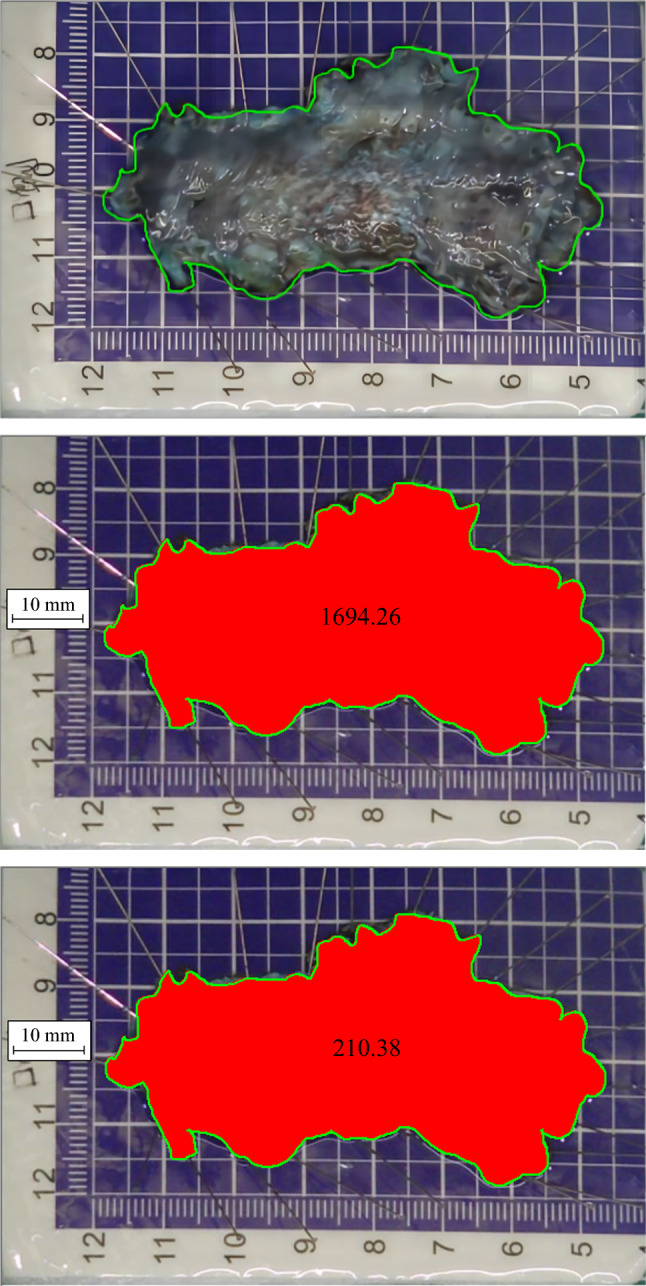
Measurement of the area and perimeter of the resected esophageal mucosa defect; **A** irregular AOI function of Image Pro Plus 6.0 software was used to outline the measurement area (green line); **B** red region represents the area of resected mucosa (1694.26 mm^2^ in this case); **C** green line represents the perimeter of the resected mucosa (210.38 mm in this case); *Note*: The actual scale of each grid on the background specimen plate was 5 mm

The area and perimeter of the specimens were measured using the Image Pro Plus 6.0 program (MediaCybernetics, Silver Spring, MD, USA), as shown in Fig. 1A–C. The circumferential ratio of mucosal defects was estimated by reviewing the documented ratio of the lesion in the operation record. Circumferential or near circumferential defects are denoted as “1.” Defects that cover half of the circumference are denoted as “0.5,” those that cover two-thirds are denoted as “0.67”, and so forth (values equal to or less than one-fifth of the circumference were consistently recorded as “0.2”). The Image Pro Plus 6.0 program was utilized to measure the area and perimeter of the mucosal defect within the near-circumferential defect, where the mucosal bridge was preserved during ESD. For a circumferential defect where the mucosal bridge was not preserved, the area was calculated by area = circumference × longitudinal length. Since the mucosa obtained from a circumferential resection was cylindrical, calculating the circumference of the upper and lower sides could not accurately reflect its larger resection area. Therefore, these cases were not included in the perimeter statistics. The assessment of MPI during ESD procedure was conducted using the MPI scale.^9^ (grade 0: no defected injury, class i: injury without full perforation, and grade p: injury with full perforation). MPI and the presence or absence of mucosal bridges during ESD were converted into categorical variables (absence denoted as 0, presence denoted as 1, both MPI-i and MPI-p denoted as 1).

#### Specimen Fixation Method

The esophageal ESD specimens were secured on a graduated specimen board and photographed. To minimize tension-induced variability during specimen pinning, the specimen fixation method uniformly follows the steps below: (1) delicately rinse the specimen’s surface to eliminate blood and mucus after ESD immediately; (2) lay the specimen on a sterile gauze lightly dampened with normal saline to ensure it remains moist; (3) with forceps or a fine needle, carefully smooth out the wrinkles from the edge, taking care not to overstretch the tissue; and (4) prioritize pinning the oral and anal sides, then carefully continue unfolding along the perimeter while preserving the specimen’s inherent tension. Secure the specimen using insect pins placed approximately 1–2 mm from the edge, with a pin spacing of around 5–10 mm. Trained technicians followed this protocol to ensure reproducibility, mitigating measurement bias.

### Statistical Analysis

Logistic regression analysis was conducted with SPSS 27.0 software to ascertain independent risk factors for esophageal stenosis subsequent to ESD. Diagnostic efficacy was evaluated using receiver operating characteristic (ROC) curves. The area under the ROC curve (AUC) was used to evaluate the predictive value. The measurement data that followed a normal distribution were expressed as mean ± SD, and a *t*-test was conducted to compare the two groups. The counting data were presented as frequency (%), and comparisons were conducted using either the chi-squared test or Fisher’s exact test. Spearman correlation analysis was employed to examine the correlation between variables. The prediction model was constructed using the R software (version 4.2.2), and the rms package was utilized to generate nomogram visuagraphs for obtaining variable score criteria. The discriminatory and calibration capabilities of the model were evaluated using the concordance statistic (C-statistic) and the Hosmer–Lemeshow goodness-of-fit (GOF) test. *P* < 0.05 was considered statistically significant.

## Results

### General Information

Finally, 614 patients were included, of whom 6.68% (41/614) had stenosis and 11.73% (72/614) had MPI as a complication. There were 78 patients (78/614) who underwent mucosal resection for more than two-thirds of the esophageal circumference. Of these, 64 patients had resection for more than two-thirds to nearly the full circumference, while 14 patients underwent a fully circumferential resection. The antistenosis effect of prophylactic steroids treatment is presented in Supplementary Table 1. Subsequent analysis focused only on individuals who did not have prophylactic antistenosis treatment, and baseline characteristics of the 561 enrolled patients is presented in Table 1. Median size of the resected mucosal area was 527.74 mm^2^ (IQR 322.80–823.48), median perimeter was 91.75 mm (IQR 71.69–118.32), and median circumferential ratio was 33% (IQR 20–40). According to Paris classification, endoscopic lesion morphology was classified as follows: polypoid (type I), nonpolypoid (type IIa: superficial, elevated; IIb: flat; IIc: superficial shallow, depressed), mix type: any two types of endoscopic lesions mentioned above coexist. According to the distribution of skeletal muscle and smooth muscle in the esophageal muscularis propria, the distance from the onset of esophageal mucosal lesions to the central incisor was divided into three groups: ≤ 17cm (skeletal muscle region), 18–25 cm (transitional region), and ≥ 26 cm (smooth muscle region).^10^ There were statistically significant differences in age and MPI grade between the stenosis and nonstenosis groups (all* P* < 0.05). However, no significant differences were found in gender, endoscopic lesion morphology, and lesion starting location (all* P* > 0.05).

**Table 1:** Baseline characteristics of enrolled patients

Characteristics	Nonstenosis	Stenosis	*P*-value
Age (years, mean ± SD)	62.07 ± 8.83	65.70 ± 8.33	0.022
*Gender [n (%)]*			0.139
Male	362 (68.6%)	18 (54.5%)	
Female	166 (31.4%)	15 (45.5%)	
*MPI scale [n (%)]*			0.002
MPI-0	470 (89.0%)	24 (72.7%)	
MPI-i	57 (10.8%)	7 (21.2%)	
MPI-p	1 (0.2%)	2 (6.1%)	
*Origin of lesion [n (%)]*			0.097
≤ 17 cm	6 (1.1%)	1(3.0%)	
18–25 cm	123 (23.3%)	12 (36.4%)	
≥ 26 cm	399 (75.6%)	20 (60.6%)	
*Endoscopic lesion morphology [n (%)]*			0.153
I	4 (0.80%)	1 (3.0%)	
IIa	37 (7.0%)	5 (15.2%)	
IIb	469 (88.8%)	26 (78.8%)	
IIc	14 (2.7%)	1 (3.0%)	
Mix type	4 (0.8%)	0 (0.00%)	

*MPI* muscularis propria injury

### Analysis of Risk Factors for Stenosis

Univariate analysis: a positive correlation was observed between postoperative stenosis and the perimeter (OR 1.035, 95% CI 1.025–1.045), area (OR 1.002, 95% CI 1.001–1.003,* P* < 0.001), circumferential ratio (OR 847.815, 95% CI 129.982–5529.898, *P* < 0.001), and MPI (OR 0.329, 95% CI 0.146–0.742, *P* = 0.007) of mucosal defect after ESD.

Multivariate variable analysis: there was a collinear relationship between the perimeter and the area of the mucosal defect (*r* = 0.958, *P* < 0.001); therefore, they were analyzed separately. When the mucosal defect area was included, the mucosal defect area (OR 1.001, 95% CI 1.001–1.002, *P* < 0.001) and the circumferential ratio (OR 89.016, 95% CI 9.856–803.976, *P* < 0.001) were identified as independent risk factors for postoperative stenosis (Table 2). When the mucosal defect perimeter was included, the mucosal defect perimeter (OR 1.033, 95% CI 1.021–1.046, *P* < 0.001) was an independent risk factor for postoperative stenosis, while the circumferential ratio (OR 3.498, 95% CI 0.155–78.713, *P* = 0.430) was not (Table 2). There was no statistically significant difference in MPI (*P* > 0.05).

**Table 2:** Results of multivariate logistic regression analysis

Variable	OR (95% CI)	*P*-value	Variable	OR (95% CI)	*P*-value
Area	(1.001–1.002)	< 0.001	perimeter	(1.021–1.046)	< 0.001
Circumferential ratio	(9.856–803.976)	< 0.001	Circumferential ratio	(0.155–78.713)	0.430
MPI	(0.188–1.309)	0.157	MPI	(0.193–1.818)	0.360

*MPI* muscularis propria injury, *OR* odds ratio, *CI* confidence interval

### ROC Curve Analysis and Establishment and Validation of Nomogram Model

The dataset was partitioned into a training set and a validation set using a random allocation ratio of 7:3. There were no statistically significant disparities observed in the circumferential ratio, area, perimeter of mucosal defect, and intraoperative MPI between the two groups (all *P* > 0.05). Owing to collinearity between the mucosal defect’s area and perimeter, we analyzed these separately along with the circumferential ratio and intraoperative MPI. We then developed a nomogram model from the training set to predict the risk of postESD esophageal stenosis. In the training set, the C-statistic of perimeter and area was 0.875 and 0.830, while in the validation set it was 0.747 and 0.835, respectively (Supplementary Fig. 1–2). The Hosmer–Lemeshow GOF test yielded nonsignificant results in both the training set (*χ*^2^ = 10.576 and 10.721, respectively) and the test set (*χ*^2^ = 7.001 and 6.958, respectively) (*P* > 0.05). This suggested that the prediction model demonstrated excellent discrimination and calibration. When the mucosal defect area reached 1200–1250 mm^2^ and the perimeter was 55–60 mm, the prediction score was approximately equal to that of the mucosal defect at 3/4 esophageal circumference (Supplementary Fig. 3, https://linwei1.shinyapps.io/dynnomapp; Supplementary Fig. 4, https://linwei2.shinyapps.io/dynnomapp). When the cumulative score reaches 70, there is a high probability (> 50%) of esophageal stenosis.

## Discussion

The occurrence of esophageal stenosis after ESD is strongly associated with the extent of circumferential mucosal defects,^11,12^ and the OR in this study was 847.815 (*P* < 0.001). When there is a large area of esophageal mucosal defect, the wound becomes more vulnerable to exposure from food and the reflux of acidic or alkaline liquids. This heightened exposure can intensify irritation from both mechanical and chemical sources, potentially exacerbating the inflammatory response.^13^ When damage to the esophageal lining is extensive, the processes of pathological repair involve not only the regeneration of the surrounding normal epithelium, but also the maturation of granulation tissue into fibrous connective tissue, and the transformation of resting fibroblasts into highly contractile myofibroblasts.^14,15^ Both autologous^16,17^ and allogeneic transplant^18^ of epithelial cell sheets, as well as the use of tissue engineering biomaterials^19,20^ to cover post-ESD wounds, have been proven effective in reducing fibrosis and inflammation. In addition, they minimize wound contraction after ESD.^18,19,21^ Nieponice et al.^22^ discovered that utilizing the porcine bladder extracellular matrix scaffold effectively prevented stenosis following circumferential mucosal resection of the esophagus in experimental dogs. When compared with the control group, this scaffold promoted better local epithelialization, lessened inflammation, and decreased scar tissue formation.

Owing to the immature technology of biomaterials used to cover the wound surface, currently, in the ESD procedure for nearly circumferential esophageal lesions, a small amount of normal or relatively normal mucosa is typically retained longitudinally to form a mucosal bridge in clinic. Alternatively, a minimal incision is made along the edge of irregular lesions to maximize the preservation of mucosa. Compared with circumferential mucosal resection or extended resection for irregular lesions, this method reduces the area of the mucosal defect, but it also increases the duration and complexity of the operation. Simultaneously, the perimeter of the wound with equivalent area is enlarged. Epithelial cells initiate proliferate and migrate from the edge of the defective mucosa,^23^ whereas myofibroblasts play a role in the epithelialization process.^24^ An expanded wound perimeter may enhance the effective proliferative edge, potentially accelerating epithelial repair. However, an extended perimeter of the wound edge results in a higher density of myofibroblasts per unit area, leading to a more pronounced degree of wound contraction. Several studies have suggested that the removal of multiple mucosal lesions can increase the risk of esophageal stenosis,^12,25^ which may be related to the increase in the circumferential ratio of mucosal defects around the esophagus. It remains unknown whether the increase in total perimeter of mucosal lesion resulting from multiple mucosal resections is a risk factor for esophageal stenosis after ESD. Our observation indicated that the sensitivity and specificity of the perimeter of mucosal lesions in predicting stenosis after ESD were superior compared with the area of the mucosal defect and circumferential range. In this study, there were only a few instances of circumferential mucosal lesions, and it was not yet possible to confirm whether preserving the mucosal bridge could reduce the risk of stenosis. Therefore, it remains to be determined whether increasing the complexity of the procedure to preserve a small amount of mucosal bridge is beneficial in preventing postoperative stenosis in the mucosa of nearly circumferential, irregular, or multiple adjacent lesions. Further clarification needs to be obtained through well-controlled animal models or large-scale clinical observations.

The existing literature suggested that postoperative stenosis following esophageal ESD is significantly associated with age,^26^ MPI,^4,7^ and the absence of prophylactic antistenosis^27^ after extensive mucosal resection, which was consistent with our univariate analysis results. While there is no consensus on the precise administration of postoperative corticosteroids, including timing and dosage, most studies, including our own, suggest that the oral and/or local injection of corticosteroids can help prevent esophageal stenosis after ESD.^28,29^ This study found that preventative intervention did not yield a statistically significant improvement in cases involving the resection of circumferential mucosa. However, it is important to note that this conclusion should be interpreted with caution owing to the limited sample size, and further larger prospective studies are necessary for more clarity. Aging is a significant risk factor for fibrotic disease, which can be exacerbated by changes in lipoprotein and lipid metabolism.^30^ Research has demonstrated a significant increase in the incidence of stenosis following esophageal ESD in patients aged over 65 years.^26^ At present, there is no unified risk stratification method to consider aging as a risk factor in esophageal stenosis, and the confounding factors that accompany aging are also complex and heterogeneous. This biological variation could potentially distort facts when expressed simply as continuous variables. Therefore, age was not included in the multivariate analysis in this study. Multiple studies have proposed that MPI and its degree during ESD are associated with postoperative esophageal stenosis.^4,7^ However, multivariate regression analysis revealed no significant association with MPI, contrasting with previous findings. Potential confounding factors, such as mucosal defect size obscuring MPI effects, warrant further investigation. Operations such as myotomy and myographic tumor enucleation using tunnel endoscopy technology could potentially cause significant harm to the submucosa and muscularis propria of the esophagus, while the occurrence of postoperative stenosis is rare.^31^ In Liu et al.’s^32^ animal investigation, it was discovered that experimental pigs, from which only the mucosal ceiling of a submucosal tunnel in the esophagus was removed, displayed esophageal stenosis and shortening. This contrasted with the control group, where no mucosa was removed. Thus, MPI should be considered a secondary factor affecting esophageal stenosis after ESD, with the primary factor likely being the degree of mucosal defects.^32^

The impact of the lesion’s location in the proximal or distal esophagus on postoperative stenosis remains uncertain. Some studies indicated that the upper esophageal lumen had a smaller width, which increased the likelihood of stenosis following ESD.^11,26,33^ However, the segmentation techniques used in these investigations lack consistency. There is no observed connection between the location of the lesion and the occurrence of postoperative stenosis, as shown by our study and previous studies.^11^ It is worth noting that existing literature has not paid attention to the potential relationship between the muscle type of the muscularis propria of the esophagus and stenosis. The histological analysis of the esophagus by Meyer et al.^10^ found that 5% of the muscle layer in the proximal end of the esophagus was dominated by skeletal muscle, 50–60% of the distal end was dominated by smooth muscle, and the middle part was a mixed transitional zone of the two types of muscle. Accordingly, we classified the starting sites of the lesions. However, we found no significant impact of the muscle type distribution at the injury site on the occurrence of postoperative esophageal stenosis (*P* = 0.097) following ESD. It should be noted that the distance from the incisors in our esophageal segmentation method significantly differed from that reported in previous studies. Given the small sample size of the subgroup and the uncertainty of estimating muscle distribution endoscopically on the basis of the distance from the incisors, further viviperception is needed to ascertain whether different types of muscle layer in the mucosal resection area impact postoperative esophageal stenosis.

Given the limitations of using lesion length and circumferential ratio alone to predict postoperative stenosis after esophageal ESD, we employed image analysis technology to measure both the area and perimeter of mucosal defects, providing a more comprehensive assessment. Unlike traditional rough measurement methods used in previous studies, we achieved accurate measurement of the resected mucosal area through image analysis technology, and for the first time, explored the potential influence of the resected mucosal perimeter on esophageal stenosis. In our multivariate analysis, the perimeter of resected mucosa was a stronger predictor than the circumferential ratio, which lost its significance as an independent risk factor. We developed a nomogram model on the basis of mucosal loss and muscularis propria injury, which yielded highly accurate results and closely matched observed probabilities, aiding in the identification and timely intervention for high-risk patients. Among the 41 patients with esophageal stricture in our cohort, preoperative lesions involved ≤ 1/2 of the luminal circumference in 20 cases and ≤ 1/3 in 12 cases, none of whom received postoperative prophylactic antistricture measures. Of the patients with lesions involving > 3/4 of the circumference, 35 developed no postoperative strictures, including 7 who underwent no prophylactic interventions. These results indicated that the circumferential extent of mucosal defects following esophageal ESD alone did not reliably predict postoperative stenosis. Existing clinical protocols may fail to identify some high-risk cases. Our model improved risk stratification by integrating both defect area and perimeter measurements, enhancing predictive accuracy and clinical utility.

There are certain limitations in this study. In this retrospective study, the inclusion of patients with esophageal stenosis following ESD was much lower than the number of patients without stenosis, which might introduce bias in the findings. The approximate values of mucosal defect area and perimeter were measured objectively by image analysis software. However, the measurement of the circumferential ratio of mucosal defects was determined on the basis of the lesion range, which was susceptible to subjective factors. Nevertheless, considering that the actual mucosal resection range extended slightly beyond the range affected by the lesion, it was reasonable to believe that the actual diagnostic performance of estimated circumferential ratio in predicting esophageal stenosis was inferior compared with the overall assessment of the lesion’s area and perimeter. The suboptimal performance of the perimeter in the validation set could undermine the reliability of our findings. Only internal validation was performed owing to the retrospective nature of the study. External validation and stronger model performance would necessitate prospective studies with larger cohorts.

In conclusion, mucosal defect area and perimeter were independent risk factors for post-ESD esophageal stenosis and were not inferior predictors when compared with the circumferential ratio. Large, irregular mucosal lesions increase stenosis risk. A predictive model that comprehensively assessed the resected mucosal area and perimeter may assist in determining the best prevention strategies.

## Supplementary Information

Below is the link to the electronic supplementary material.

Supplementary file1 (DOCX 223 KB)

## Data Availability

The data that support the findings of this study are available from the first author, Wei Lin, upon reasonable request.
