# Supplementary material for: Endoscopic Image-Based Prediction of Esophageal Stenosis after ESD Using Mucosal Defect Metrics
Source: Ann Surg Oncol. 2025 Aug 16;32(12):9262–9. doi: 10.1245/s10434-025-18037-7 (PMC12534364; doi:10.1245/s10434-025-18037-7)
Supplement: Supplementary file 1 — Supplementary file1 (DOCX 223 KB) [file 10434_2025_18037_MOESM1_ESM.docx]

**supplementary Figure 1** ROC curve analysis circumferential ratio, perimeter of mucosal defect and intraoperative MPI

**supplementary Figure 2** ROC curve analysis of circumferential ratio, area of mucosal defect and intraoperative MPI

**supplementary Figure 3** Nomogram model including circumferential ratio, area of mucosal defect and intraoperative MPI

**supplementary Figure 4** Nomogram model including circumferential ratio, perimeter of mucosal defect and intraoperative MPI

**supplementary Table 1** The antistenosis effect of prophylactic steroids treatment

**
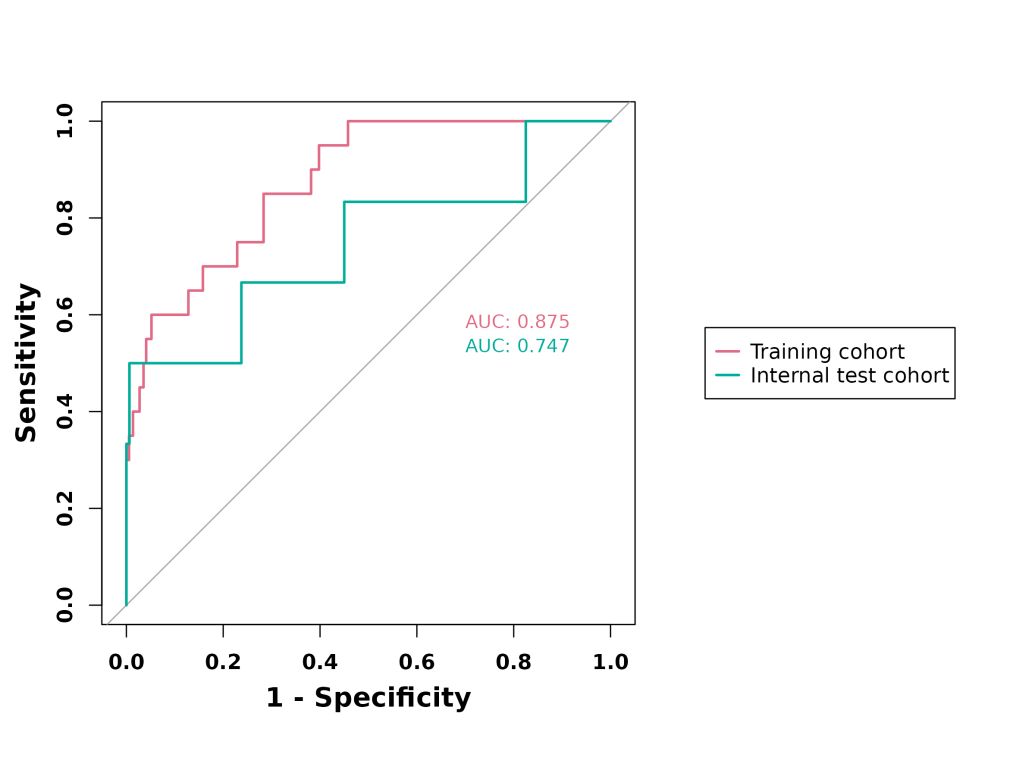
**

supplementary Figure 1


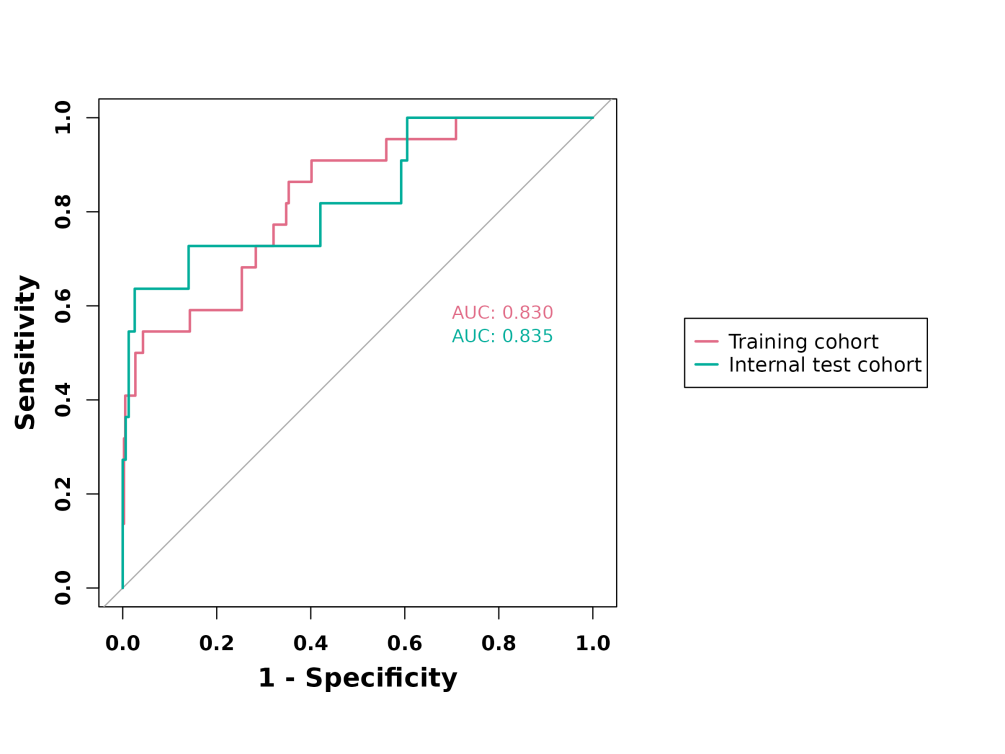


supplementary Figure 2


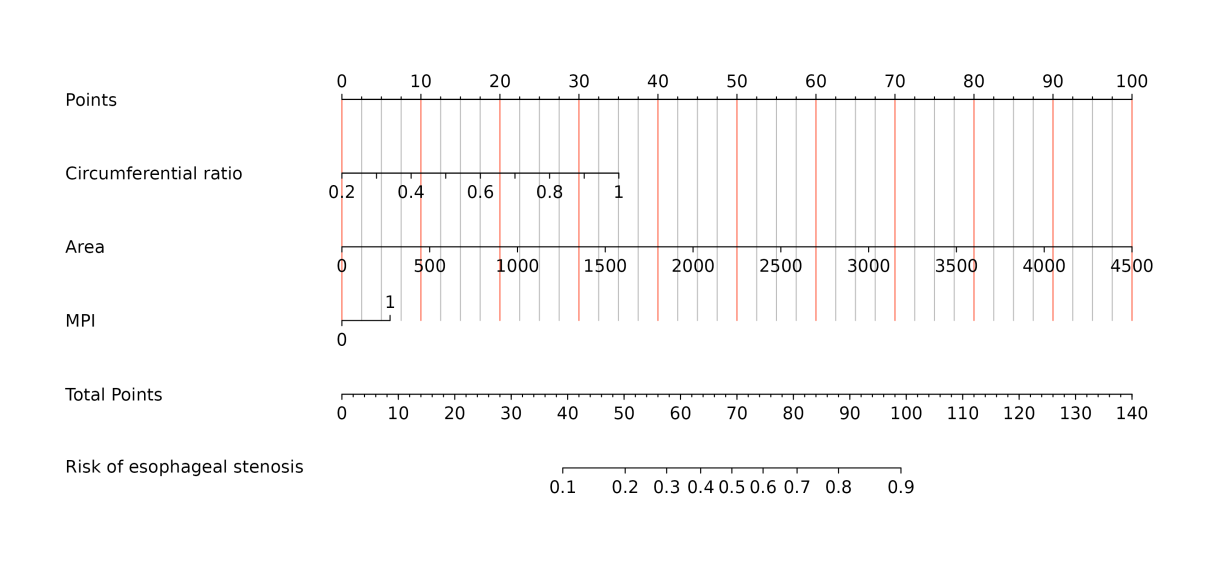
supplementary Figure 3


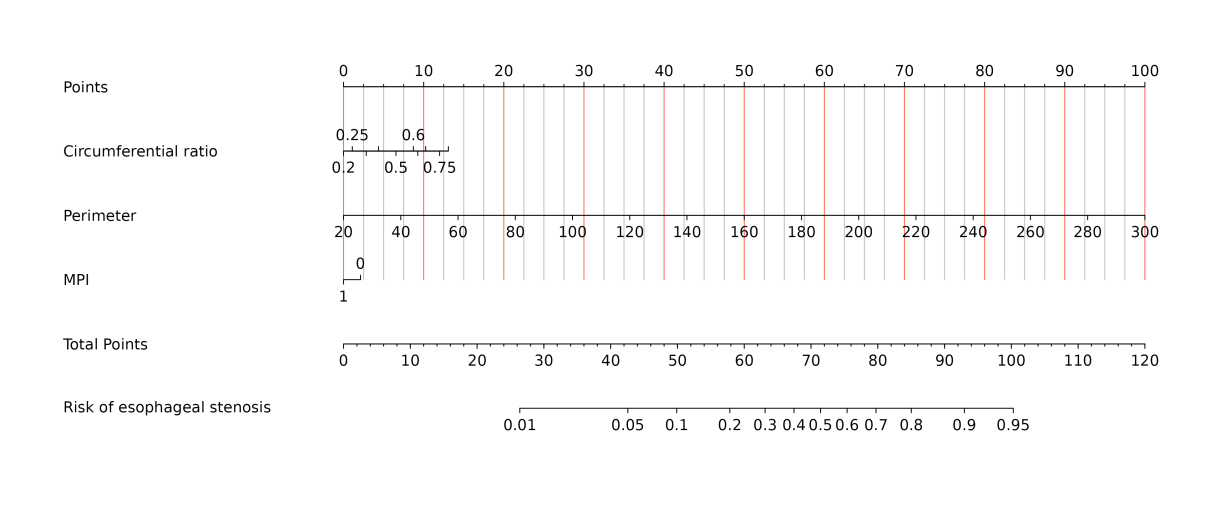


supplementary Figure 4

| Circumferential ratio | Non-stenosis | Stenosis | p-value |
| --- | --- | --- | --- |
| Over two-thirds[n (%)] |  |  | < 0.001 |
| With prophylactic antistenosis | 45(80.36%) | 11(19.64%) |  |
| Without prophylactic antistenosis | 8 (36.37%) | 14(63.63%) |  |
| Between two-thirds and near circumferential[n (%)] |  |  | 0.008 |
| With prophylactic antistenosis | 44(86.3%) | 7(13.7%) |  |
| Without prophylactic antistenosis | 10(52.6%) | 9 (47.4%) |  |
| Entire circumferential[n (%)] |  |  | 0.464 |
| With prophylactic antistenosis | 1(50.0%) | 1(50.0%) |  |
| Without prophylactic antistenosis | 1(16.7%) | 5 (83.3%) |  |

supplementary Table 1
